# Monocular Vision-Based Pose Determination in Close Proximity for Low Impact Docking

**DOI:** 10.3390/s19153261

**Published:** 2019-07-24

**Authors:** Gangfeng Liu, Congcong Xu, Yanhe Zhu, Jie Zhao

**Affiliations:** State Key Laboratory of Robotic and Systems, Harbin Institute of Technology, Harbin 150001, China

**Keywords:** monocular vision, low impact docking, pose determination

## Abstract

Pose determination in close proximity is critical for space missions in which monocular vision is one of the most promising solutions. Although numerous approaches such as using artificial beacons or specific shapes on spacecrafts have proved to be effective, the high individuation and the large time delay limit their use in low impact docking. This paper proposes a unified framework to determinate the relative pose between two docking mechanisms by treating their guide petals as measurement objects. Fusing the pose information of one docking mechanism to simplify image processing and creating an intermediate coordinate system to solve the perspective-n-point problem greatly improve the real-time performance and the robustness of the method. Experimental results show that the position measurement error is within 3.7 mm, while the rotation error around docking direction is less than 0.16°, corresponding to a measurement time reduction of 85%.

## 1. Introduction

Low impact docking [1] is a subject of intense research in the context of current docking systems. It is widely used in on-orbit servicing (OOS) [2], comet and asteroid exploration [3,4], and active debris removal (ADR) [5]. One of its core technologies is pose determination in close proximity. Pose determination generally refers to computing the relative position and the attitude between objects. The relative pose is unambiguously identified by six degrees of freedom (DOFs)—three DOFs for the relative position and three DOFs for the relative attitude. For low impact docking, pose determination occurs over a distance of less than several meters (depending on the size of the target), and its ultimate goal is to obtain the relative pose between the docking mechanisms of two spacecrafts (either cooperative or non-cooperative) with high speed, precision, and robustness. Most current methods are indirect, measuring the relative pose between two spacecrafts and then calculating the relative pose between two docking mechanisms by using the assembly relation between spacecraft and docking mechanism.

Over the last decades, state-of-the-art techniques and algorithms have been developed for cooperative and uncooperative pose determination by electro-optical (EO) sensors [6]. EO sensors have a low power consumption and can be used to estimate all pose parameters. Consequently, such sensors are the preferred instruments for this application. In general, EO sensor systems can be classified as passive systems, systems consisting of single (monocular) or multiple (stereo) cameras, and active light detection and ranging (LIDAR) systems. Among these systems, monocular vision systems have the lowest hardware complexity and cost and can be used for remote monitoring. A stereo vision system uses more than one camera, enabling it to acquire three-dimensional (3D) information about the target. However, monocular and stereo vision systems suffer from the same handicaps as all vision systems—sensitivity to illumination conditions and difficulty segmenting objects from complex backgrounds. In contrast, LIDAR is robust to differences in illumination and can obtain both position and intensity data in 3D; however, a LIDAR system consumes more energy and exhibits poorer real-time performance due to its enormous computational burden and high complexity. Thus, after weighing the pros and cons of the various methods, many research institutions and scholars have chosen to focus on pose determination based on monocular vision.

A typical pose determination method usually relies on artificial beacons that are accurately mounted on the target spacecraft. The proximity operation sensor (PXS) designed by the National Space Development Agency of Japan (NASDA) for the 7^th^ mission of the Engineering Test Satellite Program (ETS-VII) [7,8] consists of a camera and an array of light-emitting diodes (LEDs) on the chaser and a set of passive markers on the docking interface of the target. The required installation precision of the markers is very high, and the installation process usually requires high-precision measuring equipment. During the docking phase (relative distance m), the LEDs emit pulsed visible light (at a wavelength of 640 nm) within a cone of 30° to illuminate the docking interface. Simultaneously, the camera captures images that contain the markers. Then, the data processing unit calculates the relative pose using a complex image processing algorithm. The experimental results represent the advanced performance of the present method, i.e., the measurement frequency of the PXS is 2 Hz, with centimeter-scale accuracy in the relative position and one-tenth-of-a-degree-scale accuracy in the relative attitude [9]. Similar to the PXS, the advanced video guidance sensor (AVGS) [10,11] designed by the Marshall Space Flight Center and the visual based system (VBS) [12,13,14] designed by the Technical University of Denmark both require artificial beacons, which are either passive markers (reflectors) or active markers (LEDs). Sansone et al. and Pirat et al. similarly determined the pose of CubSats by using a camera and LED marks [15,16]. However, in general, these methods can be used for cooperative spacecraft pose determination only.

Another widely used method utilizes the specific shape of the spacecraft for pose determination. Liu and Hu proposed a new architecture for estimating the relative poses of cylindrical spacecrafts [17]. However, this method is not suitable for spacecrafts of other shapes. Du et al. presented a collaborative camera system for determining the pose of a large, non-cooperative satellite based on a rectangular feature [18]. Similarly, Zhang et al. determined the pose of non-cooperative spacecrafts by employing the rectangular structure of a solar panel [19]. However, this system may have risks in terms of reliability. The illumination in the space environment strongly influences this system. Moreover, if one of the cameras is compromised, the system will not function. Gao et al. designed a monocular structured light vision system for large, non-cooperative satellites [20]. However, this method is suitable only for satellites with rectangular features on their antennae, and, similar to the case of artificial beacons, the antenna needs to be accurately mounted in a specific location.

In this paper, we present a novel and efficient monocular vision system that is suitable for the low impact docking. To overcome the dependence on artificial beacons or specific shape of spacecrafts and reduce the measurement uncertainty, we utilize the guide petals of the docking mechanisms as the measurement objects. Additionally, above all, fusing the pose information of one docking mechanism to simplify image processing and creating an intermediate coordinate system to solve the perspective-n-point (PnP) problem greatly improve the real-time performance and the robustness of the system. Other improvements include the design of active light sources that minimize the sensitivity to the illumination in the space environment and the development of an effective and robust algorithm for multitarget tracking and pose determination.

This paper is organized as follows. The coordinate systems used in this paper and a comparison of the measurement uncertainties of the different methods are introduced in Section 2. Subsequently, we introduce the architecture of the monocular vision system and the design of the active light source in Section 3. Section 4 details the core algorithms of the monocular vision system. Then, the results of experiments using a ground-based, semi-physical simulation platform are reported in Section 5. Finally, we draw conclusions about the proposed method in Section 6.

## 2. Problem Formulation

To make the subsequent description and derivation process clearer and easier to understand, we first define the relevant coordinate systems in the first part of this section. Then, the rest of this section presents a comparison of the pose determination methods and theoretically proves the superiority of the proposed method.

### 2.1. Definition of the Coordinate Systems

**Figure 1:**
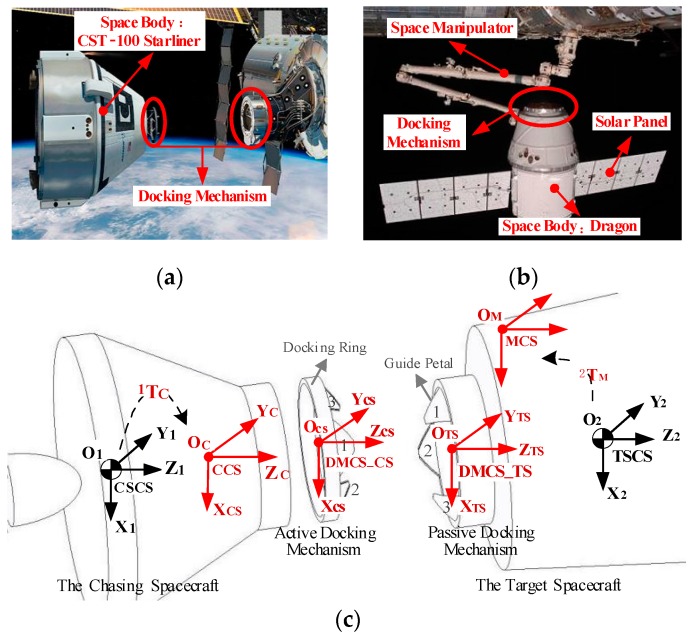
Coordinate systems defined in this paper. (**a**) CST-100 Starliner developed by Boeing (docking mechanisms are marked with red circles). (**b**) Dragon developed by SpaceX (docking mechanism is marked with a red circle). (**c**) Definitions of the coordinate systems.

Here, we define the coordinate systems used in this paper: the camera coordinate system (CCS), the docking mechanism coordinate system of the chasing spacecraft (DMCS_CS), the docking mechanism coordinate system of the target spacecraft (DMCS_TS), the mark coordinate system (MCS), the chasing spacecraft coordinate system (CSCS), and the target spacecraft coordinate system (TSCS). In general, a spacecraft consists of the spacecraft body, the docking mechanism, a camera or marks, and other equipment, such as a pair of long solar panels, an antenna, and/or a space manipulator (see Figure 1a,b). The defined coordinate systems are shown in Figure 1c and described in Table 1. When defining the CCS, we assume that the camera is an ideal camera, namely, that its principal point coincides with the center of the image and that it has zero skew and an aspect ratio of 1. The origin O_C_ of the CCS is located at the optical center of the camera, and its distance from the center of the image is the focal length f. The Z_C_ axis coincides with the camera’s optical axis and points to the object being measured. The X_C_ and Y_C_ axes are parallel to the X and the Y directions of the imaging plane, respectively. The homogeneous variation matrix of the CCS in the CSCS is TC1. Note that the MCS represents the coordinate system of either the artificial beacons or the specific shape of the spacecraft used to determine the target pose, as mentioned above. Therefore, only the homogeneous variation matrix TM2 of the MCS in the TSCS is given.

### 2.2. Comparison of Different Methods

No measurement is exact, as is well known. When a quantity is measured, the outcome depends on the measuring equipment, the measurement procedure, the environment, and other factors. Based on different types of measurement objects, we distinguish two possible measurement methods. For the first method (M1), the measurement objects are artificial beacons or specific spacecraft shapes. For the second method (M2, our proposed method), the measurement objects are the guide petals of the docking mechanisms. In the introduction, we explain the limitations of current methods. In order to further evaluate the performance of different measurement methods, we compare the uncertainty, the error, and the frequency. Under the same conditions, the same EO sensor and assembly accuracy, the measurement quality of the methods can be evaluated by comparing the measurement uncertainty. The lower the measurement uncertainty is, the better the quality is. The detailed process is described as follows.

First, expressions for the relative poses between the docking mechanisms under the two methods are derived via homogeneous transformation as follows, where Equation (1) is for M1 and Equation (2) is for M2:

(1) TTSCS=T1CS⋅TC1⋅TMC⋅T2M⋅TTS2,

and

(2) TTS′CS=TCCS⋅TCSC.

Refer to the coordinates shown in Figure 1c, TBA represents the homogeneous transformation matrix from coordinate system A into coordinate system B. It is a 4 × 4 square matrix consisting of a rotation matrix RBA and a translation matrix PBA, i.e., TBA=[RBAPBA01]. Suppose that N is the quantity to be measured and that x,y,z… are direct measurements, such that N=f(x,y,z…). From the measurement uncertainty formula, one can obtain the measurement uncertainty of N as follows:

(3) $$\sigma_{N}=\sqrt{{\left(\frac{\partial f}{\partial x}\right)}^{2}\sigma_{x}^{2}+{\left(\frac{\partial f}{\partial y}\right)}^{2}\sigma_{y}^{2}+{\left(\frac{\partial f}{\partial z}\right)}^{2}\sigma_{z}^{2}+\ldots }.$$

Then, by substituting Equations (1) and (2) into Equation (3), we can obtain the measurement uncertainty formulas for the two methods, as shown in Equations (4) and (5). For M1, the measurement uncertainty formula is:

(4) $$\sigma =\sqrt{{\left(\frac{\partial f}{\partial x_{1}}\right)}^{2}\sigma_{x_{1}}^{2}+{\left(\frac{\partial f}{\partial y_{1}}\right)}^{2}\sigma_{y_{1}}^{2}+{\left(\frac{\partial f}{\partial z_{1}}\right)}^{2}\sigma_{z_{1}}^{2}+{\left(\frac{\partial f}{\partial w_{1}}\right)}^{2}\sigma_{w_{1}}^{2}+{\left(\frac{\partial f}{\partial u_{1}}\right)}^{2}\sigma_{u_{1}}^{2}},$$

where x1=T1CS, y1=TC1, z1=TMC, w1=T2M and u1=TTS2. For M2, the measurement uncertainty formula is:

(5) $$\sigma^{\prime }=\sqrt{{\left(\frac{\partial f}{\partial x_{2}}\right)}^{2}\sigma_{x_{2}}^{2}+{\left(\frac{\partial f}{\partial y_{2}}\right)}^{2}\sigma_{y_{2}}^{2}},$$

where x2=TCCS and y2=TTSC. $\sigma_{x_{1}}$, $\sigma_{y_{1}}$, $\sigma_{w_{1}}$, and $\sigma_{u_{1}}$ (the measurement uncertainties of T1CS, TC1, T2M, and TTS2, respectively) are mainly due to $E_{1}$ (manufacturing error and assembly error); thus, it is reasonable to assume that $\sigma_{x_{1}}=\sigma_{y_{1}}=\sigma_{w_{1}}=\sigma_{u_{1}}$. $\sigma_{z_{1}}$ and $\sigma_{y_{2}}$ (the measurement uncertainties of TMC and TTSC, respectively) are mainly due to $E_{2}$ (measurement error from pose determination); thus, $\sigma_{z_{1}}=\sigma_{y_{2}}$. For $\sigma_{x_{2}}$ (the measurement uncertainty of TCCS), we choose the source with the least error: $\sigma_{x_{2}}=\{\begin{array}{cc} \sigma_{x_{1}}=\sigma_{y_{1}}=\sigma_{w_{1}}=\sigma_{u_{1}}, & E_{1}\leq E_{2}\\ \sigma_{Z_{1}}=\sigma_{y_{2}}, & E_{1}>E_{2} \end{array}.$ Therefore, $\sigma >\sigma^{\prime }$. Finally, the results can be summarized as follows:

- As shown in Equations (1) and (2), our proposed method M2 works well for pose measurement for both cooperative and non-cooperative targets, and it is much simpler and more efficient than the existing method M1.
- The measurement accuracy of M2 is higher than that of M1 since $\sigma >\sigma^{\prime }$.

## 3. Design of the Monocular Vision System

We designed a monocular vision system for determining the relative pose between two docking mechanisms for low impact docking. This system, especially the active light source, is described in detail in this chapter.

### 3.1. Architecture of the Monocular Vision System

**Figure 2:**
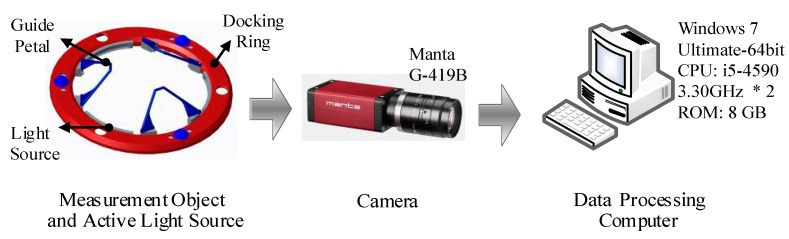
The framework of the monocular vision system.

**Figure 3:**
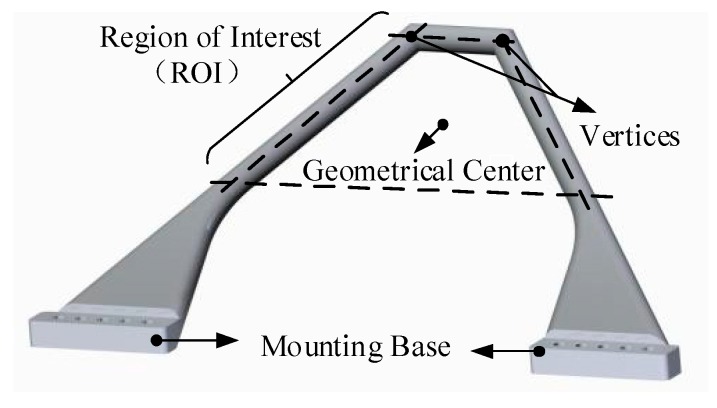
The vertices of a guide petal and geometrical center of the region of interest (ROI).

The monocular vision system consists of three parts, namely, an active light source, a camera, and a data processing computer, as shown in Figure 2. The active light source is mounted on the docking ring of the active docking mechanism. It moves with the docking ring to provide active illumination; its detailed structure is introduced in Section 3.2 below. Because of the short measuring distance, the small range of movement of the docking mechanism and the high measurement accuracy required, we chose the Manta G-419B camera produced by Allied Vision Technologies. The physical resolution is 2048 × 2048 pixels, and the cell size is 5.5 µm. The maximum frame rate at full resolution is 28.6 fps, and the lens’ theoretical focal length is 8 mm. As mentioned above, the camera is installed inside the docking mechanism, i.e., on the hatches (see Figure 1), and will not affect the normal passage of astronauts and cargo. The camera periodically captures images and transmits them to the data processing computer via a gigabit Ethernet (GigE) interface. Then, the data processing computer performs image processing and pose calculation to obtain the relative position between the docking mechanisms. In the method proposed in this paper, the spacecraft as a whole is not the measurement object; instead, only the vertices of the guide petals (see Figure 3) are measured. Therefore, the monocular vision system is designed to capture a clear image of the guide petals.

### 3.2. Design of the Active Light Source

**Figure 4:**
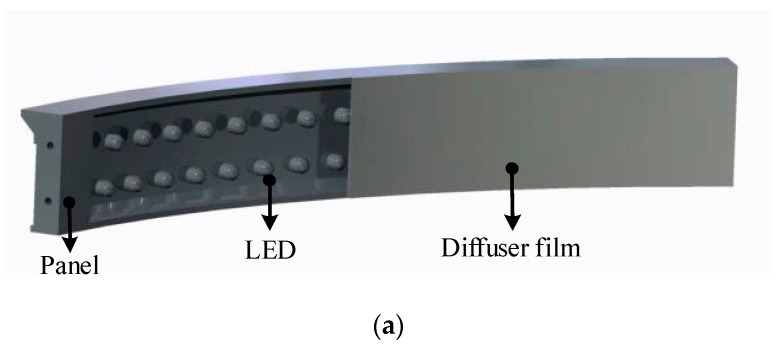

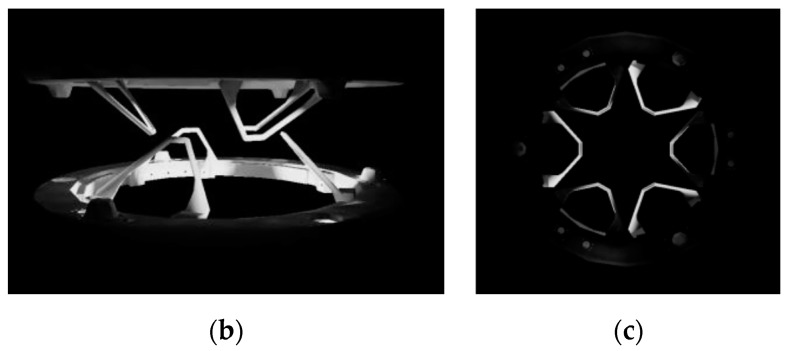
Active light source. (**a**) The internal structure of the arc-shaped light-emitting diode (LED) panel. (**b**) A rendering of the active light source (front view). (**c**) A rendering of the active light source (side view).

In a vision system designed to determine the relative pose for low impact docking in a complex space environment, illumination becomes a key factor. It is particularly difficult to maintain the objects to be measured under suitable illumination conditions. The typical, simple method is to use an integrating sphere as a uniform source to illuminate the target. Then, all objects in the field of view have a similar grayscale range. However, segmenting the target object from the background requires complex image processing algorithms, and this complexity seriously affects the stability and the real-time performance of the system. Considering this restriction, a distributed active light source was designed, which can adaptively adjust both the brightness and the illuminated area. The active light source consists of three arc-shaped LED panels that are mounted at even intervals on the inner wall of the active docking ring (see Figure 2) and move with the active docking ring to accommodate changes in the relative pose. During the close-proximity docking process, only the guide petals of the two docking mechanisms are illuminated. Each LED panel consists of a panel, multiple LEDs, and a diffuser film (see Figure 4a). Because of the diffuser film, the light from the LED point sources is diffused into 120 degrees of diffuse light. Thus, the guide petals are uniformly illuminated, while the surrounding objects are not, as shown in Figure 4b,c. Hence, the active light source ensures that the guide petals remain under suitable lighting conditions throughout the docking process.

## 4. Key Algorithms of the Monocular Vision System

**Figure 5:**
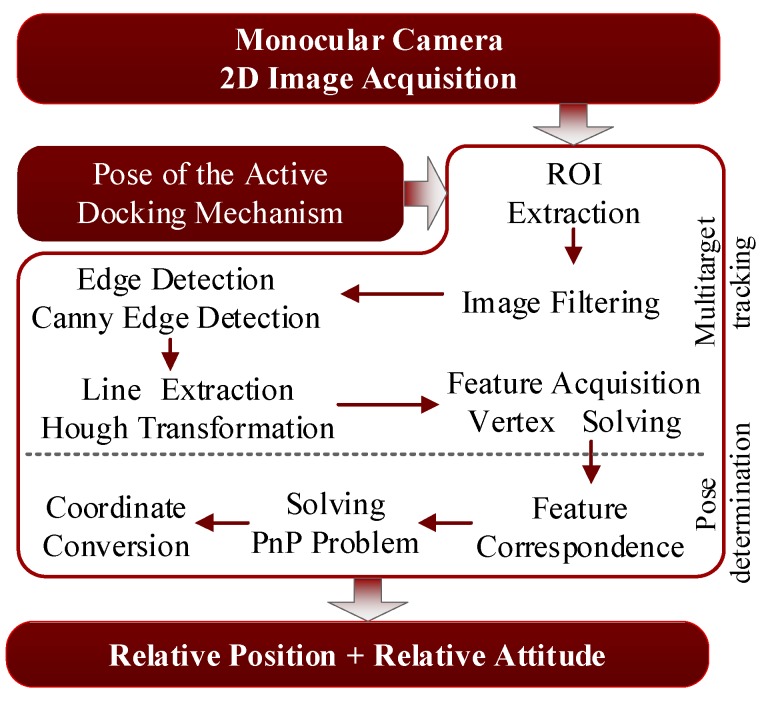
Algorithmic framework of the monocular vision system.

The algorithmic framework for monocular vision-based pose determination is shown in Figure 5. It has two key components: multitarget tracking and pose determination. The details of these steps are presented in Section 4.1 and Section 4.2.

### 4.1. Multitarget Tracking

Because of the complexity of the space environment, the structure of the target spacecraft and the imaging characteristics of a monocular vision system with a high original image resolution (2048 × 2048), it is challenging to design a monocular vision system with high performance, low algorithm complexity, and insensitivity to the pose and the geometry of the target spacecraft. To solve these problems, we introduce the pose information of the active docking mechanism for multitarget tracking.

#### 4.1.1. ROI Extraction

At the beginning of the low impact docking process, the relative pose between the docking mechanisms is within a certain range, as described by the initial docking conditions. To achieve low impact docking, the pose of the active docking mechanism must be adjusted during the docking process. The regions of interest (ROIs), namely, the areas corresponding to the guide petals in the image, are related to the pose of the active docking mechanism. There are six square ROIs in the image during the docking process; we define the center coordinates of each ROI as $P_{i}^{roi}=(u_{i},v_{i})\;(i=1,\ldots ,6)$ and the length of each ROI as $d^{roi}$ (in pixels). The derivation process of $P_{i}^{roi}$ and $d^{roi}$ is as follows.

**Figure 6:**
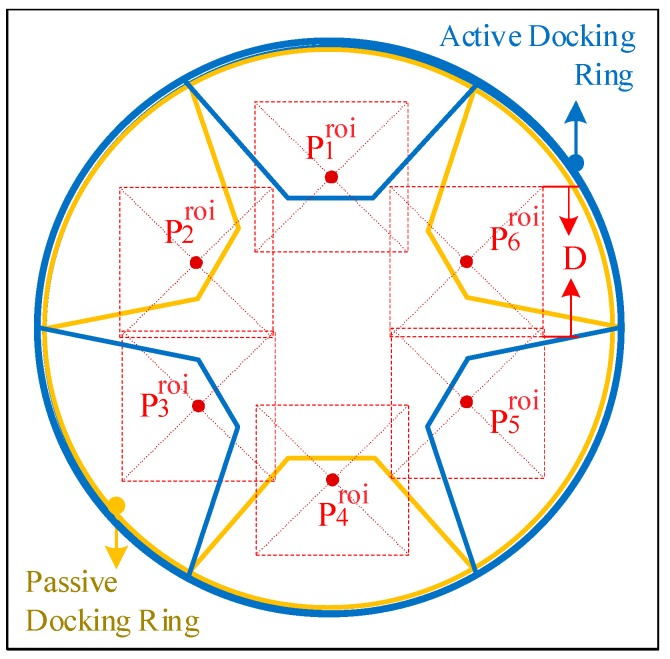
A simplified diagram of the camera viewpoint when the active docking mechanism is in the initial position.

As shown in Figure 3, the coordinates PiCS=(XiCS,YiCS,ZiCS,1)T (i=1,…,6) denote the geometrical center of each guide petal, and D is the true length corresponding to $d^{roi}$ (the side length of each square ROI). P1CS, P3CS, and P5CS are located on the active docking mechanism, and P2CS, P4CS, and P6CS are located on the hypothetical passive docking mechanism, as shown in Figure 6. TCSidealC is the ideal homogeneous transformation matrix for the CCS to the DMCS_CS, and it is not an exact value. $T^{CS}$ is the homogeneous transformation matrix of the active docking mechanism, i.e., the pose information. By applying a homogeneous transformation, PiCS can be transformed into the CCS to obtain PiC=(XiC,YiC,ZiC,1)T (i=1,…,6):

(6) PiC=TCSidealC⋅TCS⋅PiCS (i=1,…,6).

Substituting PiCS into Equation (6), we obtain Equation (7) as follows:

(7) [XiCYiCZiC1]=TCSidealC⋅TCS⋅[XiCSYiCSZiCS1] (i=1,…,6).

Suppose that the camera’s interior and external parameters are K and T. The three-dimensional point $\left(X_{W},Y_{W},Z_{W}\right)$ in the world coordinates can map to the two-dimensional pixel point (u,v):

(8) $$\left[\begin{array}{c} u\\ v\\ 1 \end{array}\right]=K\cdot T\cdot \left[\begin{array}{c} X_{W}\\ Y_{W}\\ Z_{W}\\ 1 \end{array}\right].$$

After calibration, we can obtain the interior parameter. The external parameter is related to TCSidealC and $T^{{}_{CS}}$. Thus, PiCS can be obtained as follows:

(9) [uiroiviroi1]=[kx0u00kyv0001]⋅TCS⋅[XiCSYiCSZiCS1] (i=1,…,6).

In addition, since the rotations of the docking mechanisms during the docking process are relatively small, $d^{roi}$, the length of each ROI in pixels, is mainly related to the distance of the active docking mechanism:

(10) $$\frac{f}{d^{roi}}=\frac{Z_{i}^{C}}{L}\;(i=1,\ldots ,6).$$

Then,

(11) $$d^{roi}=\frac{L}{Z_{i}^{C}}\cdot f\;(i=1,\ldots ,6).$$

**Figure 7:**
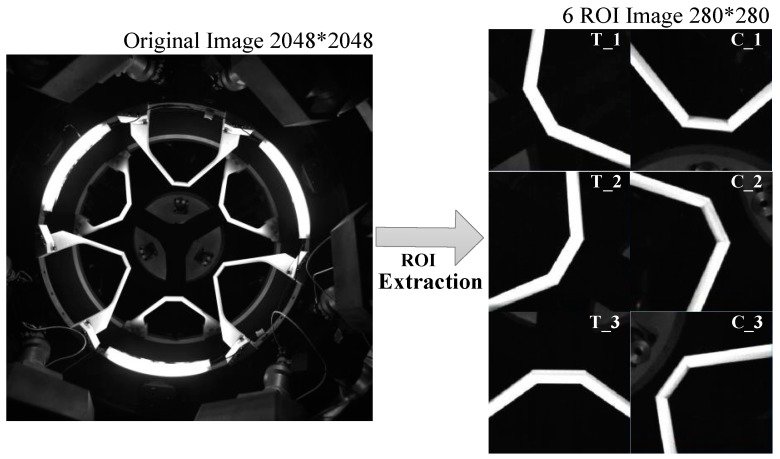
Six subimages acquired based on the pose information of the active docking mechanism.

Thus, using ROI extraction, we can track the measurement objects accurately and rapidly, as shown in Figure 7.

#### 4.1.2. Image Processing

After the ROI extraction, we detect the twelve vertices of the six guide petals with a series of image processing algorithms. The steps of this algorithm include image filtering, edge detection, line extraction, and feature acquisition.

**Figure 8:**
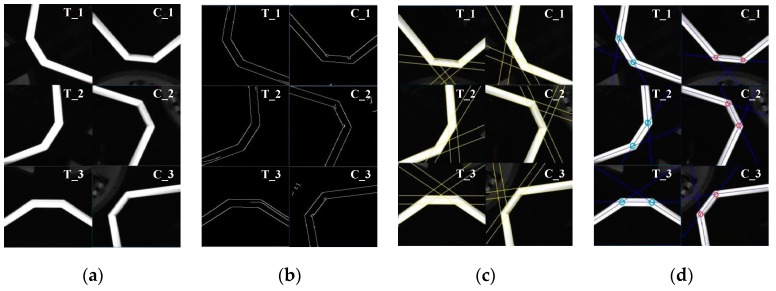
Image processing for multitarget tracking. (**a**) Image filtering. (**b**) Edge detection. (**c**) Line extraction. (**d**) Feature acquisition.

In general, noise is introduced into a visual system, and this noise can contaminate the images acquired by the system. It is necessary to filter out the noise in an image before edge detection. For image filtering, the most common methods include normalized box filtering, Gaussian filtering, median filtering, and bilateral filtering. To balance computational speed and filtering performance, we choose median filtering [21]. Median filtering is a nonlinear image smoothing technique that sets the gray value of each pixel to the median value among all pixels in a selected neighborhood. This technique can protect the edges in an image such that they are not blurred when the noise is filtered out. The mathematical expression of the median filtering process is as follows:

(12) g(x,y)=med{f(x−k,y−i),(k,i∈W)},

where f(x,y) and g(x,y) are the original and the processed images, respectively, and W is a two-dimensional template. This template usually has square dimensions of 3 × 3 or 5 × 5; alternatively, it can be a different shape, such as a line, circle, or cross. Figure 8a illustrates the effect of median filtering.

For edge detection, the Canny operator is well known as an adaptable and efficient operator [21]. Hence, it is used in this paper to detect the edges of the guide petals. The Canny edge detection algorithm includes four calculation steps: first, Gaussian smoothing is performed; next, the gradient value and the direction of the first-order differential partial derivative are calculated; then, the non-maximum extreme value is suppressed; and finally, the edge connection is created using a dual threshold. In this way, we obtain the edges of the six guide petals, as shown in Figure 8b.

Binary images containing the edges of the guide petals are obtained after the application of the Canny algorithm. To recognize the linear edges of the targets, the Hough transform [21] is used. A prominent advantage of this approach is its robustness due to its insensitivity to data inaccuracies and noise. The Hough transform maps the points in an image from Cartesian space into polar coordinates. More specifically, N curves that intersect at the same point in polar space correspond to N points on the same straight line in Cartesian space. Figure 8c shows the line extraction results achieved using the Hough transform.

To resolve the vertices of the guide petals, we first calculate the centerline of each side of each guide petal. In polar coordinates, the line corresponding to one edge of one side of a guide petal is represented by $\left(\theta_{L},r_{L}\right)$, and the line corresponding to the other edge is represented by $\left(\theta_{R},r_{R}\right)$. Here, $\theta_{i}$ represents the polar path, and $r_{i}$ represents the polar angle (i=L/R). Then, the centerline is represented by (θ,r):

(13) $$\{\begin{array}{l} \theta =\frac{\theta_{L}+\theta_{R}}{2}\\ r=\frac{r_{L}+r_{R}}{2} \end{array}.$$

Therefore, in Cartesian space, the corresponding straight line equation is as follows:

(14) cosθ⋅u+sinθ⋅v+r=0.

To calculate the intersection between two such centerlines, we assume that the two lines are represented by $\left(\theta_{1},r_{1}\right)$ and $\left(\theta_{2},r_{2}\right)$. According to Equation (13), we can establish the following linear equations:

(15) $$\left[\begin{array}{cc} \cos\theta_{1} & \sin\theta_{1}\\ \cos\theta_{2} & \sin\theta_{2} \end{array}\right]\left[\begin{array}{c} u\\ v \end{array}\right]=\left[\begin{array}{c} r_{1}\\ r_{2} \end{array}\right],$$

where (u,v) denotes the intersection coordinates, representing the vertex of the guide petal, in Cartesian space. Thus, (u,v) can be solved for as follows:

(16) $$\{\begin{array}{l} u=\frac{\sin\theta_{2}r_{1}-\sin\theta_{1}r_{2}}{\cos\theta_{1}\sin\theta_{2}-\sin\theta_{1}\cos\theta_{2}}\\ v=\frac{\cos\theta_{2}r_{1}-\cos\theta_{1}r_{2}}{\sin\theta_{1}\cos\theta_{2}-\cos\theta_{1}\sin\theta_{2}} \end{array}.$$

Finally, we obtain the twelve vertices of the six guide petals, as shown in Figure 8d.

### 4.2. Pose Determination

The next stage is to resolve the relative pose, which includes feature correspondence, the solution of the perspective-n-point problem, and coordinate conversion. 

#### 4.2.1. Feature Correspondence

For feature correspondence, namely, 3D-2D (two-dimensional) point matching, the 2D feature points are mapped to 3D feature points according to the angular ranges of the centerlines. For example, ROI subimage $T_{1}$ has three centerlines, $L_{1}$, $L_{2}$, and $L_{3}$, and their ranges are $L_{1}\in \left[1.0,\;2.1\right]$, $L_{2}\in \left[2.2,\;2.9\right]$, and $L_{3}\in \left[0,\;0.5\right]\cup \left[3.0,\;3.14\right]$ (radians, in polar space). Therefore, the 2D image intersections of $L_{1}-L_{2}$ and $L_{2}-L_{3}$ correspond to the vertices of the corresponding guide petal.

#### 4.2.2. Solution of the PnP Problem

The solution of the PnP problem is the most important and difficult step of this part. There are many algorithms available to solve the PnP problem, such as P3P, EPnP [22] and UPnP [23]. However, each algorithm has its restrictions. For example, P3P limits the input perspective points to 4, EPnP requests that the perspective points be non-coplanar, and UPnP’s calculations are rather complex. To calculate the relative pose efficiently and robustly, an indirect solution using the intermediate coordinate system is proposed. The following derivation process is to determine the relative position of the docking mechanism coordinate system of the chasing spacecraft (DMCS_CS) to the camera coordinate system (CCS).

After the previous processing, the projections of the six vertices of DMCS_CS in the normalized image plane can be obtained, which are $p_{n}=(u_{n},v_{n})$. Given the limitations of the docking initial conditions, the docking process can obtain at least four and up to six vertices. That is, the maximum values of n are 4, 5, and 6.

**Figure 9:**
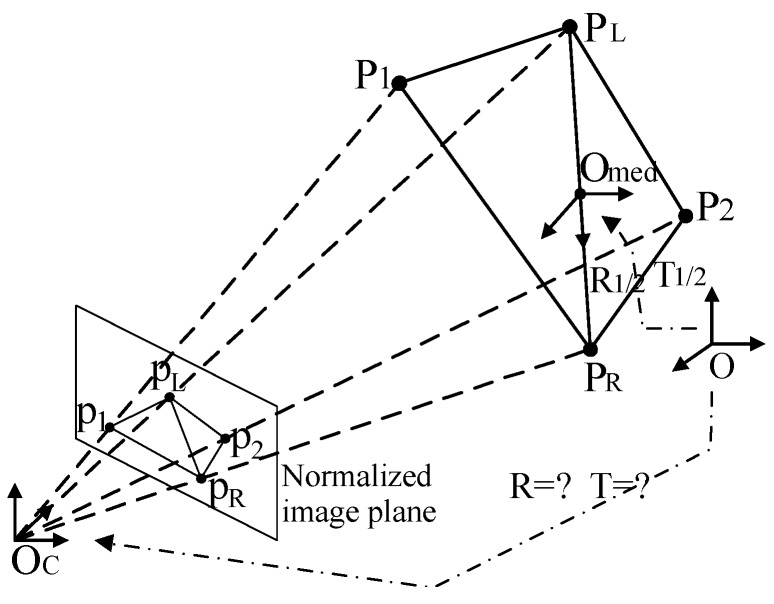
The projections of the vertices.

To create an intermediate coordinate system O_med_X_med_Y_med_Z_med_, the longest projection length $\left\|P_{L}P_{R}\right\|$ is selected, as shown in Figure 9. Then, we use the vector $\vec{P_{L}P_{R}}$ as the rotation axis X_med_, and the origin is at the center of $\bar{P_{L}P_{R}}$. Inspired by a robust solution to the perspective-n-point problem (RPnP) [24], we divide the n vertices into three-point subsets such as $\left\{P_{L}P_{R}P_{k}|n\neq L,n\neq L,k\in \{1\ldots n\}\right\}$. The constraint of each subset yields one polynomial of order 4 as follows:

(17) $$\{\begin{array}{l} f_{1}(x)=a_{1}x^{4}+b_{1}x^{3}+c_{1}x^{2}+d_{1}x+e_{1}=0\\ f_{2}(x)=a_{2}x^{4}+b_{2}x^{3}+c_{2}x^{2}+d_{2}x+e_{2}=0\\ \ldots \\ f_{n-2}(x)=a_{n-2}x^{4}+b_{n-2}x^{3}+c_{n-2}x^{2}+d_{n-2}x+e_{n-2}=0 \end{array}.$$

By using the least-squares residual, a cost function $F=\sum_{i=1}^{n-2}f_{i}^{2}(x)$ is defined as the square sum of Equation (17). The minimum of F can be obtained by solving $F^{\prime }=\sum_{i=1}^{n-2}f_{i}(x)f_{i}^{\prime }(x)=0$. As soon as x is determined, the vertices in the CCS can be calculated, and $X_{med}=\vec{P_{L}P_{R}}/\left\|P_{L}P_{R}\right\|$. Then, the rotation matrix from the intermediate coordinate system to the CCS can be expressed as:

(18) $$R_{med}=R^{0}rot(X,\alpha )=\left[\begin{array}{ccc} r_{1} & r_{4} & r_{7}\\ r_{2} & r_{5} & r_{8}\\ r_{3} & r_{6} & r_{9} \end{array}\right]\left[\begin{array}{ccc} 1 & 0 & 0\\ 0 & c & -s\\ 0 & s & c \end{array}\right],$$

where $R^{0}$ is an arbitrary orthogonal matrix and ${\left[r_{7}\;r_{8}\;r_{9}\right]}^{T}$ equals the rotation axis X_med_. rot(X,α) denotes a rotation α of degrees around X_med_, with s=sin(α) and c=cos(α).

The projection from the 3D points in the intermediate coordinate system to the 2D normalized image plane can be expressed as follows:

(19) $$\lambda_{n}\left[\begin{array}{c} u_{n}\\ v_{n}\\ 1 \end{array}\right]=\left[\begin{array}{ccc} r_{1} & r_{4} & r_{7}\\ r_{2} & r_{5} & r_{8}\\ r_{3} & r_{6} & r_{9} \end{array}\right]\left[\begin{array}{ccc} 1 & 0 & 0\\ 0 & c & -s\\ 0 & s & c \end{array}\right]\left[\begin{array}{c} X_{n}^{med}\\ Y_{n}^{med}\\ Z_{n}^{med} \end{array}\right]+\left[\begin{array}{c} t_{x}\\ t_{y}\\ t_{z} \end{array}\right].$$

where $t={\left[tx\;ty\;tz\right]}^{T}$ is the translation vector. Rearranging Equation (19), we have:

(20) $$\left[\begin{array}{ccc} A_{2n+1} & B_{2n*1} & C_{2n*4} \end{array}\right]\left[\begin{array}{c} c\\ s\\ t_{x}\\ t_{y}\\ t_{z}\\ 1 \end{array}\right]=0,$$

where $A_{2n*1}=\left[\begin{array}{c} r_{6}Y_{1}^{med}u_{1}+r_{9}Z_{1}^{med}u_{1}-r_{4}Y_{1}^{med}-r_{7}Z_{1}^{med}\\ r_{6}Y_{1}^{med}v_{1}+r_{9}Z_{1}^{med}v_{1}-r_{5}Y_{1}^{med}-r_{8}Z_{1}^{med}\\ \ldots \\ \ldots \\ r_{6}Y_{n}^{med}u_{n}+r_{9}Z_{n}^{med}u_{n}-r_{4}Y_{n}^{med}-r_{7}Z_{n}^{med}\\ r_{6}Y_{n}^{med}v_{n}+r_{9}Z_{n}^{med}v_{n}-r_{5}Y_{n}^{med}-r_{8}Z_{n}^{med} \end{array}\right],$ $B_{2n*1}=\left[\begin{array}{c} r_{9}Y_{1}^{med}u_{1}-r_{6}Z_{1}^{med}u_{1}-r_{7}Y_{1}^{med}+r_{4}Z_{1}^{med}\\ r_{9}Y_{1}^{med}v_{1}-r_{6}Z_{1}^{med}v_{1}-r_{8}Y_{1}^{med}+r_{5}Z_{1}^{med}\\ \ldots \\ \ldots \\ r_{9}Y_{n}^{med}u_{n}-r_{6}Z_{n}^{med}u_{n}-r_{7}Y_{n}^{med}+r_{4}Z_{n}^{med}\\ r_{9}Y_{n}^{med}v_{n}-r_{6}Z_{n}^{med}v_{n}-r_{8}Y_{n}^{med}+r_{5}Z_{n}^{med} \end{array}\right]$ and $C_{2n*1}=\left[\begin{array}{cccc} -1 & 0 & u_{1} & r_{3}X_{1}^{med}u_{1}-r_{1}X_{1}^{med}\\ 0 & -1 & v_{1} & r_{3}X_{1}^{med}v_{1}-r_{2}X_{1}^{med}\\ \ldots & \ldots & \ldots & \ldots \\ \ldots & \ldots & \ldots & \ldots \\ -1 & 0 & u_{n} & r_{3}X_{n}^{med}u_{n}-r_{1}X_{n}^{med}\\ 0 & -1 & v_{n} & r_{3}X_{n}^{med}v_{n}-r_{2}X_{n}^{med} \end{array}\right].$ The unknown variable vector ${\left[c\;s\;t_{x}\;t_{y}\;t_{z}\;1\right]}^{T}$ can be retrieved by solving the linear equation system using singular value decomposition. That is, the rotation matrix and the translation vector from the intermediate coordinate system to the CCS can be obtained.

After the intermediate coordinate system is determined, we can easily obtain the rotation matrix and the translation vector from OCS to O_med_. Then, using homogeneous transformation, the rotation matrix and the translation vector, TCCS can be obtained from OCS to OC.

#### 4.2.3. Coordinate Conversion

Here, we obtain the rotation matrix and the translation vector from DMCS_CS and DMCS_TS to CCS, i.e., TCCS and TCTS, respectively. Thus, the relative pose between the two docking mechanisms is:

(21) TTS′CS=TCCS⋅TTSC=TCCS⋅(TCTS)−1.

## 5. Ground-Based Semi-Physical Simulation Experiments

**Figure 10:**
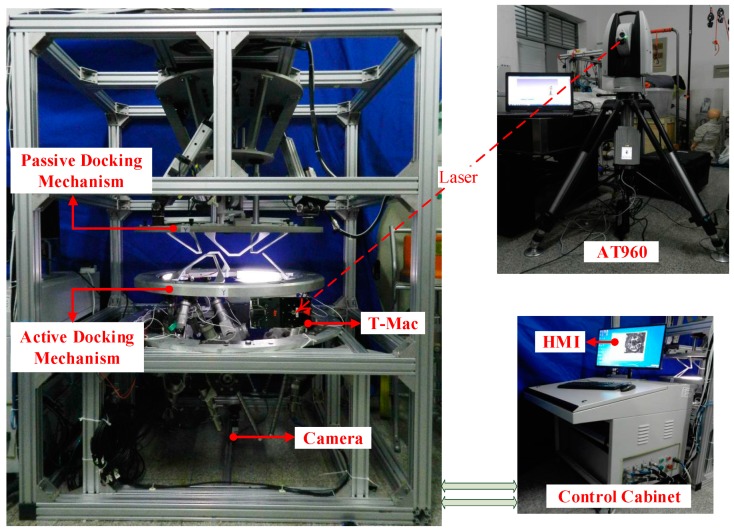
The semi-physical simulation platform.

To verify the proposed method, semi-physical simulation experiments are presented in this section. All experiments were performed with the same semi-physical simulation platform. This platform is mainly composed of an active docking mechanism (Stewart platform), a passive docking mechanism, a monocular vision system, a data processing and control cabinet, a human-machine interface (HMI), a Leica T-Mac (TMC30-B), and a Leica Absolute Tracker (AT960) (as shown in Figure 10). The active docking mechanism at the bottom of the frame is used to simulate the chasing spacecraft, and the passive docking mechanism at the top of the frame is used to simulate the target spacecraft. The structure of the monocular vision system and its installation relationship with the docking mechanism were previously described. The cabinet realizes the motion control of the docking mechanism and the data processing for the vision system. The T-Mac (TMC30-B) and the Absolute Tracker (AT960) are laser measuring devices manufactured by Leica Geosystems. The combination of the T-Mac and the Absolute Tracker enables the measurement of the six DOFs between the docking mechanisms. The corresponding measurement uncertainties are shown in Table 2.

The purpose of the experiments was to verify the proposed method under close-proximity conditions. The docking mechanisms of the platform are approximately half the size of an actual docking mechanism. Therefore, for docking in close proximity, we assume that the distance between the docking mechanisms is less than 0.1 meters. Before the experiments, we fixed the passive docking mechanism and measured its relative pose in the coordinate system of the laser tracker. Then, we fixed the active docking mechanism and measured its pose. During the experiments, the passive docking mechanism was always fixed, and the active docking mechanism was controlled to move through eight groups of specific poses. Each group consisted of 243 ($243{=3}^{5}$) poses defined by selected combinations of X, Y, Z, Rx, Ry, and Rz, and each of them had three different values, as shown in Table 3. They were not precise relative poses but rather served as input data to control the active docking mechanism.

After the active docking mechanism moved to the above poses, the monocular vision system captured images, calculated relative poses, and saved the data. At the same time, the laser tracker measured and saved the T-Mac poses. We assume that the result measured by the laser tracker is the true value of the relative pose between the docking mechanisms. Accordingly, the difference between the value measured by the monocular vision system and this true value is the measurement error of the monocular vision system. To understand the process, six typical cases are clearly shown in the Supplementary Materials.

**Figure 11:**
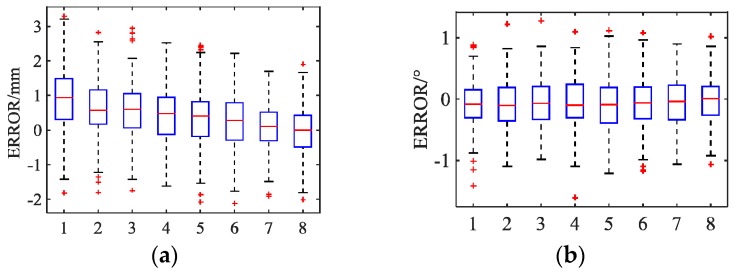

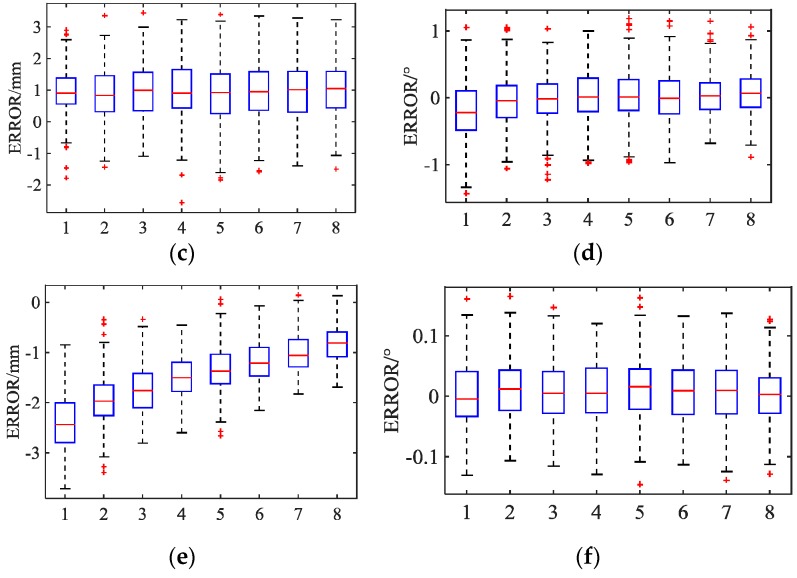
Measurement errors of the monocular vision system. (**a**) E_X_ (Groups 1–8). (**b**) E_Rx_ (Groups 1–8). (**c**) E_Y_ (Groups 1–8). (**d**) E_RY_ (Groups 1–8). (**e**) E_Z_ (Groups 1–8). (**f**) E_RZ_ (Groups 1–8).

As shown in Figure 11, the measurement errors are $E_{X}\in [-2.1,3.3]$, $E_{Y}\in [-2.5,3.4]$, $E_{Z}\in [-3.7,0.1]$, $E_{Rx}\in [-1.6,1.3]$, $E_{Ry}\in [-1.4,1.2]$ and $E_{Rz}\in [-0.15,0.16]$ (mm/°). The measurement results marked with a “+” symbol represent high noise in the measurement process, but this does not mean that the data are invalid. It is important to note that the data of each group are normally distributed due to the existence of Gaussian noise. Thus, in most cases, there is millimeter-scale accuracy in the relative position and one-tenth-of-a-degree-scale accuracy in the relative attitude. The measurement errors observed in these experiments are smaller than those of the existing measurement systems, such as PXS. Moreover, the measurement frequency is approximately 13.5 Hz; that is, the measurement time is 85% less than that of PXS (2 Hz). These results show that the proposed method is feasible and efficient.

## 6. Conclusions

This paper discusses the influence of relative pose determination for low impact docking in close proximity and analyzes the advantages and the disadvantages of various methods. A new pose determination method based on monocular vision is proposed after a theoretical consideration of the measurement uncertainty. The main contributions of this work can be summarized as follows:

- This paper proposed a unified framework for determining the relative pose between two docking mechanisms, which reduces the dependence of artificial beacons or the specific shape of the target spacecraft and the introduction of (manufacturing and assembly) error. Therefore, the novel method can be widely applied for low impact docking.
- The fusion of pose information and the optimization of the PnP problem solution greatly improve the real-time performance and the robustness of pose determination.
- The experiments verified that the method can be used to determinate the relative pose between two docking mechanisms in close proximity for low impact docking. Meanwhile the measurement accuracy and the speed of the proposed method are superior to those of the PXS. The position measurement error is within 3.7 mm, and the rotation error around the docking direction is less than 0.16°, corresponding to a measurement time reduction of 85%.

In the future, the improvement and the optimization of the hardware and software will be investigated by, for example, increasing the measurement speed using a graphics processing unit (GPU) and parallel computing. In particular, the cellular neural network [25] can be used for parallel processing of the six ROIs, which will greatly improve the efficiency of image processing.

## Figures and Tables

**Table 1:** Coordinate systems used for low impact docking (as shown in Figure 1).

Name	Abbreviation	Origin and Direction
Camera coordinate system	CCS	O_C_: the optical center of camera; +Z_C_: pointing toward the target; +Y_C_, +X_C_: respectively parallel to the image plane coordinate system.
Docking mechanism coordinate system of chasing spacecraft	DMCS_CS	O_CS_: center of docking ring; +Z_CS_: closing direction; +Y_CS_: line of symmetry through petal number 3; +X_CS_: forms a right-handed coordinate system.
Docking mechanism coordinate system of target spacecraft	DMCS_TS	O_TS_: center of docking ring; X_TS_, Y_TS_, Z_TS_: analogous to X_CS_, Y_CS_, Z_CS_.
Marks coordinate system	MCS	——
Chasing spacecraft coordinate system	CSCS	O_1_: CG ^1^ of chasing spacecraft; +Z_1_: closing direction; +Y_1_: analogous to + Y_CS_; +X_1_: forms a right-handed coordinate system.
Target spacecraft coordinate system	TSCS	O_2_: CG ^1^ of target spacecraft; X_2_, Y_2_, Z_2_: analogous to X_1_, Y_1_, Z_1_.

^1^ CG: center of gravity.

**Table 2:** Measurement uncertainties of the T-Mac ^1.^

T-Mac	Uncertainty
Accuracy of rotation angles	0.01° = 18 µm/100 mm (0.002″/ft)
Accuracy of time stamps	±5 ms
Positional accuracy (for one single coordinate, X, Y or Z)	±15 µm + 6 µm/m (±0.0006″ + 0.00007″/ft)

^1^ These measurement uncertainties are valid for a measuring time of 1 second and a totally static measurement.

**Table 3:** The relative poses between the two docking mechanisms.

Group	X/mm	Y/mm	Z/mm	Rx/°	Ry/°	Rz/°
1	−25/0/25		110	−5/0/5		
2	−25/0/25		100	−2.5/0/2.5		
3	−25/0/25		90	−2.5/0/2.5		
4	−20/0/20		80	−2/0/2		
5	−20/0/20		70	−2/0/2		
6	−15/0/15		60	−1.5/0/1.5		
7	−15/0/15		50	−1.5/0/1.5		
8	−10/0/10		40	−2/0/2		

## Supplementary Materials

The following are available online at https://www.youtube.com/watch?v=b0AEjwK9oBY, Video: MonocularVision
